# “Reverse torque of 30 Ncm applied to dental implants as test for osseointegration”—a human observational study

**DOI:** 10.1186/s40729-016-0060-4

**Published:** 2016-12-07

**Authors:** Sabrina G. Simeone, María Rios, Jeannette Simonpietri

**Affiliations:** Santa Maria University, Calle La Línea, Residencias El Parque, Piso 5, Apto B–5B. La Carlota. Municipio Sucre, Caracas, 1071 Venezuela

**Keywords:** Primary stability, Counterclockwise force, Prosthetic loading, Success, Failure

## Abstract

**Background:**

On bone implantology, stability of a dental implant is an essential clinical tool during osseointegration evaluation, as it is a reflection of the structural and functional connection between the bone and the implant.

**Methods:**

The sample was comprised by 17 patients with 40 NanoTec™ and Vellox® implants, placed on the lower jaw, under optimum conditions, after a minimum healing period of 3 months, during stage 2 surgery.

**Results:**

All 40 implants showed ideal clinical stability after the 30 Ncm reverse torque. There was absence of mobility, absence of radiolucid radiographic images, and symptomatology.

**Conclusions:**

The reverse torque is an accepted and non-invasive clinical method for early verification of initial integration, reducing the incidence of possible failure during the first year of prosthetic loading. This is the first study in humans which shows that 30 Ncm is possible, which means a greater safety for prosthetics, since prosthetic parts are turned with up to 35 Ncm.

## Background

Primary stability has been reestablished as a previous clinical requirement to achieve osseointegration. The presence of movements between the surface of the implant and the bone tissue induces a bone resorption that produces fibrointegration, in which the implant is surrounded by an interphase of soft or connective tissue, and not bone tissue [1, 2].

Strategies used to improve bone response include increasing the rugosity or the application of bioactive liners, to improve cellular adhesion and thus increase the bone-implant contact surface [3, 4].

Recently, a physical measuring test has been introduced to monitor the stability of the implant, after its healing period. Clinical perception of the stability of the dental implant is frequently related to rotational resistance during implant placement, or the application of a reverse torque [5].

However, it is unknown how much torque may be applied for testing without damaging the implant osseointegration. The available data for this comes from three volunteer implants and from animal research.

## Reverse torque in dental implants

The reverse torque test proposed by Roberts et al. in 1984, and developed by Johansson and Albrektsson in 1987 [6], is considered as a special advantage in stage 2 surgery, because it represents a definitive clinical verification of initial integration of the dental implant with the bone surface. The torque level required is commonly expressed in Newton centimeters (Ncm) [7, 8].

This way, a clinical evaluation is made of the perception of any movement of the dental implant, after a specific counterclockwise force. It represents an objective diagnostic tool, easy to apply, cheap, non-invasive, and capable of discriminating between a stable and a mobile implant, basing itself concomitantly on the evaluation of the existence of radiographic signs or symptoms, which could be relevant to predict the prognosis of the osseointegration of a dental implant.

The investigation methodologies generally found imply animal models. The amount and specificity of data reported on reverse torque as a clinical application in humans is scarce, with little scientific evidence to back it up. Only 13 implants in 2 studies have been reported in humans, using reverse torque as removal torque. In 1988, Tjellstrom et al. [9] measured the reverse torque for the removal of ten Ti implants placed on the mastoid bone. Nine of the implants were removed with a torque manometer, after 3 to 4 months of insertion. Only one implant was removed with a surgical drill, for further histological study of the bone-titanium interphase. The torque required to remove the implants varied from 26 to 60 Ncm, with a 42.7 Ncm average. On the other hand, in 1996, Sullivan et al. [5] studied three implants on a volunteer. The test was made after 6 months of healing. One of the implants of the upper mandible failed after the application of a reverse torque less than 10 Ncm. The remaining two implants remained integrated after the application of torque between 10 and 20 Ncm. The implant on the lower jaw required a reverse torque of 58 Ncm to be removed from the alveolar bone, and the upper one a 45 Ncm torque. Thus, it was concluded that a reverse torque of 20 Ncm applied to Ti dental implants seemed to be a safe and reliable method, as it represents less than 50% of the torque needed to break the bone-dental implant interphase, in bones quality type III or IV [7].

Since the study herein is not found in humans [5, 9], last phase of study, and counting on previous results from in vitro and in vivo researches with animal models on long bones (femur, tibia, mandibles) in dogs, pigs, baboons, rabbits, and even in mastoid bones [6, 9–16], which provide necessary information to design human trials, this mechanical test was performed on dental implants in humans, under ideal circumstances, with healing times ideally established, in areas with more bone density and quality, with no bone compromise, where the implants were embedded in bone, without any fenestrations or dehiscences, and with the main goal of setting the stage from an innovative, not very studied perspective, and act as a research background with a promising concept, in order to determine the clinical feasibility of the application of a 30-Ncm reverse torque to dental implants, so as to confirm their osseointegration before the prosthetic phase, through a predictable, adequate, and non-invasive clinical verification modality.

## Methods

**Fig. 1:**
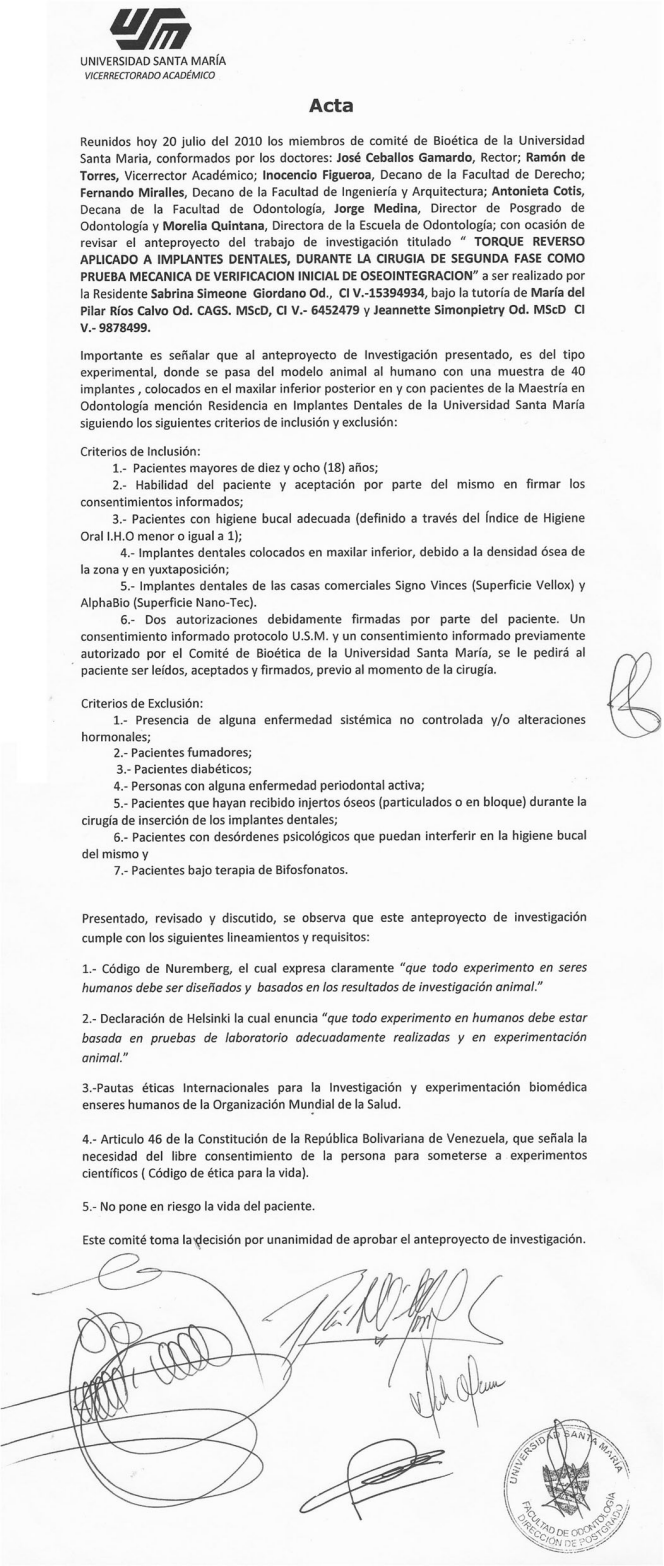
Approval of the Bioethics Committee of the Santa Maria University

A clinical trial was carried out, in which 17 patients treated with implants at the School of Dentistry of the Santa Maria University and which had had 40 dental implants placed from two different commercial brands, with NanoTec™ and Vellox® surfaces, on the lower jaw, with the approval of the Bioethics Committee of the Santa Maria University (Fig. 1).

### Inclusion criteria of the research

1. Patients 18 years old or older;
2. Signed formal written informed consent must be obtained from the patient (Fig. 2);

**Fig. 2:**
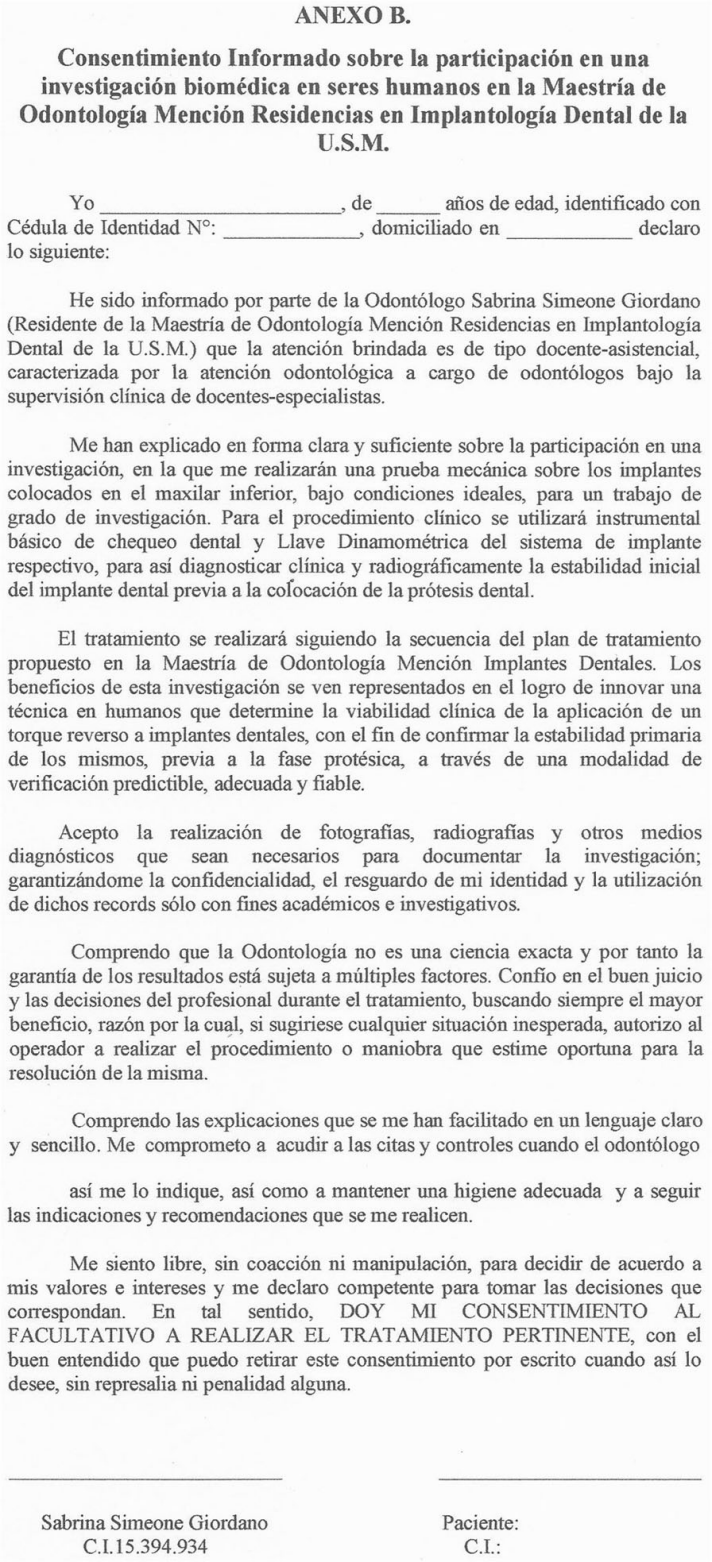
Informed consent for patients
3. Patients with proper mouth hygiene (defined through an Oral Hygiene Index of less than or equal to 1);
4. Dental implants placed on the lower jaw, due to the bone density of the area and in juxtaposition; and
5. Dental implants with Vellox® and NanoTec™ surfaces.

### Exclusion criteria of the research

1. Presence of any non-controlled systemic disease and/or hormonal disorders;
2. Smoking patients;
3. Diabetic patients;
4. People with any active periodontal disease;
5. Patients that have received bone grafts (particulate or in block) during the insertion surgery of the dental implants;
6. Patients with psychological disorders that might affect their dental hygiene; and
7. Patients under bisphosphonates therapy.

Once the patients were informed of the experimental nature of the investigation and its publication, corresponding informed consents were signed (Fig. 1), and after the pre-surgical preparation for the placement of the implants, the surgery was conducted under aseptic conditions, and in accordance with the sequence of each commercial brand. The corresponding drilling was performed with abundance of irrigation, and the drilling speed and the initial torque used for the insertion of the dental implants were recorded. After finishing the surgical procedure, periapical radiographies were taken of the area. The proper healing time was complied with, in accordance with the Brånemark Protocol (at least 3 months).

**Fig. 3:**
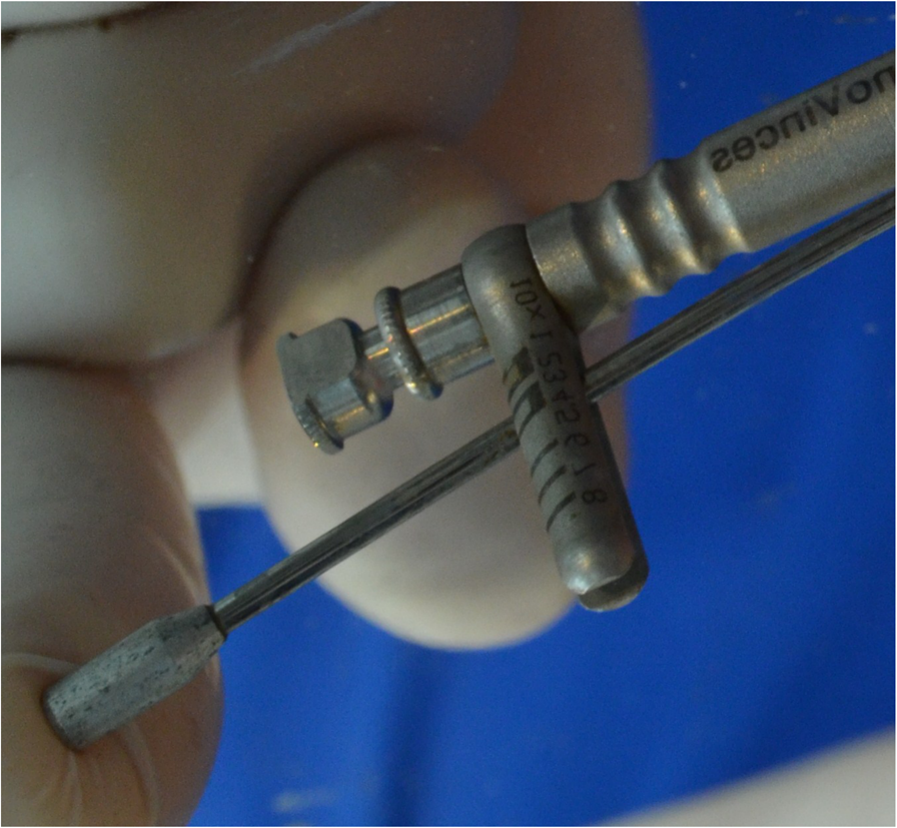
30 Ncm, measurement

**Fig. 4:**
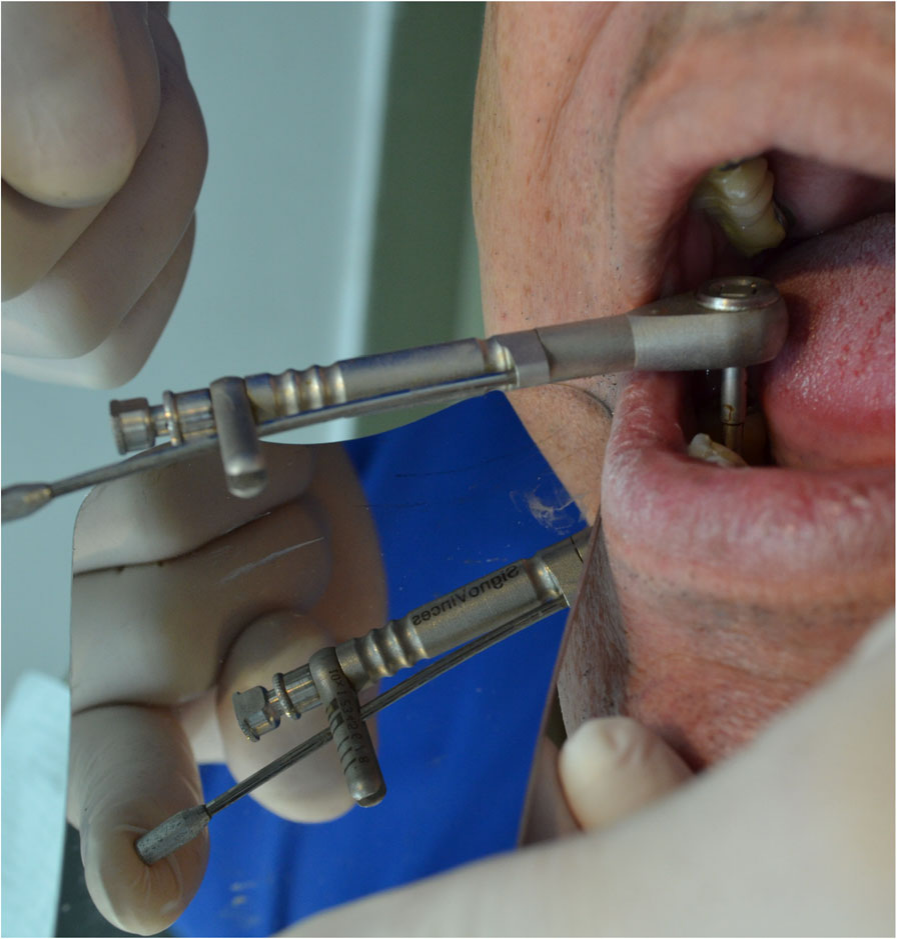
Reverse torque test

**Fig. 5:**
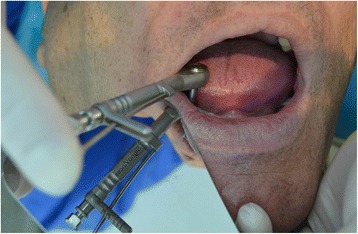
Reverse torque at 30 Ncm

Before the stage 2 surgery, periapical radiographies were taken off the area. Once the dental implant was exposed, the closing screw was removed. Before placing the healing abutment, a manual counterclockwise 30 Ncm reverse torque was applied (Fig. 3), with an Alpha Bio and/or Signo Vinces calibrated dynamometric wrench, depending on the surface of the implant placed (Figs. 4 and 5). Finally, the corresponding healing abutment was put into place, and the periapical radiography was taken.

On the data collection table, the existence of any peri-implant radiolucency radiographic images previous to the stage 2 surgery was recorded. After the application of the 30 Ncm reverse torque, the presence or absence of dental implant movement was recorded, as well as any incidence of symptomatology (pain), or presence of any clinical signs.

## Results

This study reports on the clinical behavior of 40 dental implants placed on the lower jaw, on ideal bone, and under ideal surgical and systemic conditions, subjected to a 30-Ncm reverse torque during the stage 2 surgery, on 17 patients.

The patients evaluated only showed normal signs and symptoms of post-operatory swelling. According to the distribution of the implants on the mandibular region, 2 cases of the implants with NanoTec™ surface were placed on the canine region, 9 on the premolar region, and 9 on the molar region. In the case of the implants with Vellox® surface, 6 of them were placed on the premolar region and 14 on the molar region.

Of all the implants studied, the average initial torque of insertion was 31 Ncm on the implants with NanoTec™ surface and 39 Ncm on the implants with Vellox® surface.

There was no clinical or radiographic difference between the dental implants which stage 2 surgery was performed 3 months later and those which stage 2 surgery was performed after 4 months of healing.

Clinically or radiographically, no adverse events were reported in any of the two dental implant systems during the evaluations. On the 40 dental implants subjected to a 30-Ncm reverse torque, the expected clinical integration was observed. There was no evidence of mobility, or clinical signs on the peri-implantar tissues. There was no symptomatology whatsoever, with the exception of a case on a Vellox® implant, which showed “slight” sensitivity when the reverse torque was performed.

## Discussion

The reverse torque technique proposed by Roberts in 1984 to evaluate dental implants integration proved to be successful. The most recent researches are aimed at evaluating current results. Thus, the objective of this research was to evaluate, by means of the 30 Ncm reverse torque test, 40 dental implants on the lower jaw, during the stage 2 surgery, as a mechanical and clinical, non-invasive treatment of initial osseointegration of two different implant brands, which micro and macroscopic designs are varied. The idea is to establish and contribute scientific evidence to support long-term implantological treatment plans openly, on totally or partially edentulous patients.

In accordance with authors such as Sullivan, Jividen, and Carr [5, 7, 11, 12], our main objective when performing the reverse torque test was to identify non-integrated dental implants as early as possible, before the restoration phase, through an objective method for clinical verification that is easy to perform, with readily available tools, and with a proper level of safety that will not damage the bone-dental implant interphase.

Based on previous studies, such as Carr’s in 1995 [11], who suggested, knowing the risks of data inferred from animals compared to humans, a recommended measurement of 35 Ncm when placing the prosthetic component, which has been confirmed and established by every commercial brand as the safety margin for most implants at the time of their prosthetic connection. We support our study on the application of a 30-Ncm reverse torque, as a reliable measure under the conditions of our study. Also, in accordance with the results of researches performed on reverse torque on humans, such as Sullivan’s [6], who established that a 20-Ncm reverse torque on low density bones is a reliable measurement on cone-shaped Ti implants, and authors such as Johansson and Albrektsson [6], who defined that once the dental implants are osseointegrated, the minimum reverse torque required to dislodge Ti implants with treated surface was 116 Ncm, the measurement of 30 Ncm was confirmed as a predictive measure.

In order to exclude risk factors, as there are no previous researches on reverse torque in humans, the research was made following a conventional protocol, only on lower jaw implants, with implants of treated surface, which have benefits that greatly compensate for the risks, allowing for faster osseointegration levels because the rugosity increases the contact surface between the implant and the bone tissue [13, 14]. Considering the results of our study, and the micro and macrogeometry of the implants with NanoTec™ and Vellox® surfaces, no difference was found between the two implant systems, after the 30-Ncm reverse torque.

It is worth mentioning that the reverse torque mechanical test has been the subject of very little criticism. Brånemark, in his study from 1985, argued it was a risk of irreversible plastic deformation inside the peri-implantar zone [17], if the proper healing times are not complied with, and in low density bones; however, both of these factors were excluded from our study as this is a concept test. Also, other methods, such as Periotest and the resonance frequency analysis, are more technique sensitive and depend on multiple factors, such as contact angle applied to the surface of the implant, interposition of soft tissue, malfunctioning of the device itself, or the type of implant [5, 8, 17].

## Conclusions

1. Under the ideal conditions of the study herein, the application of a 30-Ncm reverse torque at the time of the stage 2 surgery, before the prosthetic restoration, allowed the clinical identification of the stability of 40 dental implants.
2. Primary stability depends on the micro and macroscopic characteristics of the dental implant. Thus, after the application of a 30-Ncm reverse torque, both implant surfaces, NanoTec™ and Vellox®, showed the expected clinical integration.
3. Insertion torques registered did not determine any differences, due to the micro and macrogeometry of the implants studied, with respect to the 30-Ncm reverse torque.
4. Under the conditions of the study, a condition of success was clinically and radiographically established on all 40 implants evaluated, with no perceptible peri-implantar radiographic changes, clinical evidence of mobility, or presence of any symptomatology after the 30-Ncm reverse torque test.
5. Nowadays, there is a clear and demonstrable need to define a non-invasive, fast, and easy-to-use diagnostic technique to clinically evaluate the stability of a dental implant and its osseointegration, before the restoration phase. It is recommended to extend this study to other clinical cases, in order to establish application magnitudes of the reverse torque test, according to bone quality and quantity.

According to the results of this study, this clinical event was established as a mechanism for early clinical verification of osseointegration.
